# Identification of a G‐Protein Subunit‐α11 Gain‐of‐Function Mutation, Val340Met, in a Family With Autosomal Dominant Hypocalcemia Type 2 (ADH2)

**DOI:** 10.1002/jbmr.2797

**Published:** 2016-06-02

**Authors:** Sian E Piret, Caroline M Gorvin, Alistair T Pagnamenta, Sarah A Howles, Treena Cranston, Nigel Rust, M Andrew Nesbit, Ben Glaser, Jenny C Taylor, Andreas E Buchs, Fadil M Hannan, Rajesh V Thakker

**Affiliations:** ^1^ Academic Endocrine Unit, University of Oxford, Oxford Centre for Diabetes, Endocrinology and MetabolismChurchill HospitalOxfordUK; ^2^ Wellcome Trust Centre for Human GeneticsRoosevelt DriveOxfordUK; ^3^ Oxford NIHR Comprehensive Biomedical Research CentreOxfordUK; ^4^ Oxford University Hospitals NHS TrustOxford Medical Genetics LaboratoriesChurchill HospitalOxfordUK; ^5^ Sir William Dunn School of PathologyUniversity of OxfordSouth Parks RoadOxfordUK; ^6^ Biomedical Sciences Research InstituteUlster UniversityColeraineUK; ^7^ Department of Internal MedicineHadassah‐Hebrew University Medical CenterJerusalemIsrael; ^8^ Department of Medicine DAssaf Harofe Medical CenterZerifinIsrael; ^9^ Department of Musculoskeletal BiologyInstitute of Ageing and Chronic DiseaseUniversity of LiverpoolLiverpoolUK

**Keywords:** WHOLE‐EXOME SEQUENCING, G‐PROTEIN, CALCIUM, HYPOPARATHYROIDISM, KERATOCONUS

## Abstract

Autosomal dominant hypocalcemia (ADH) is characterized by hypocalcemia, inappropriately low serum parathyroid hormone concentrations and hypercalciuria. ADH is genetically heterogeneous with ADH type 1 (ADH1), the predominant form, being caused by germline gain‐of‐function mutations of the G‐protein coupled calcium‐sensing receptor (CaSR), and ADH2 caused by germline gain‐of‐function mutations of G‐protein subunit α‐11 (Gα_11_). To date Gα_11_ mutations causing ADH2 have been reported in only five probands. We investigated a multigenerational nonconsanguineous family, from Iran, with ADH and keratoconus which are not known to be associated, for causative mutations by whole‐exome sequencing in two individuals with hypoparathyroidism, of whom one also had keratoconus, followed by cosegregation analysis of variants. This identified a novel heterozygous germline Val340Met Gα_11_ mutation in both individuals, and this was also present in the other two relatives with hypocalcemia that were tested. Three‐dimensional modeling revealed the Val340Met mutation to likely alter the conformation of the C‐terminal α5 helix, which may affect G‐protein coupled receptor binding and G‐protein activation. In vitro functional expression of wild‐type (Val340) and mutant (Met340) Gα_11_ proteins in HEK293 cells stably expressing the CaSR, demonstrated that the intracellular calcium responses following stimulation with extracellular calcium, of the mutant Met340 Gα_11_ led to a leftward shift of the concentration‐response curve with a significantly (*p* < 0.0001) reduced mean half‐maximal concentration (EC_50_) value of 2.44 mM (95% CI, 2.31 to 2.77 mM) when compared to the wild‐type EC_50_ of 3.14 mM (95% CI, 3.03 to 3.26 mM), consistent with a gain‐of‐function mutation. A novel His403Gln variant in transforming growth factor, beta‐induced (TGFBI), that may be causing keratoconus was also identified, indicating likely digenic inheritance of keratoconus and ADH2 in this family. In conclusion, our identification of a novel germline gain‐of‐function Gα_11_ mutation, Val340Met, causing ADH2 demonstrates the importance of the Gα_11_ C‐terminal region for G‐protein function and CaSR signal transduction. © 2016 The Authors. *Journal of Bone and Mineral Research* Published by Wiley Periodicals, Inc. on behalf of American Society for Bone and Mineral Research (ASBMR).

## Introduction

**Figure 1:**
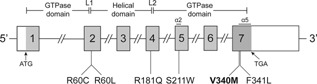
Schematic representation of the genomic organization of the human *GNA11* gene showing the location of ADH2‐causing mutations. The *GNA11* gene consists of 7 exons with the start (ATG) and stop (TGA) codons located in exons 1 and 7, respectively. The GTPase domain (encoded by exon 1, 5′ portion of exon 2, 3′ portion of exon 4 and exons 5 to 7) is connected to the helical domain (encoded by the 3′ portion of exon 2, exon 3, and 5′ portion of exon 4) by the linker 1 (L1) and linker 2 (L2) peptides. The previously reported ADH2‐causing Arg60Cys (R60C), Arg60Leu (R60L), and Arg181Gln (R181Q) mutations^3^, ^6^, ^7^ are located at the interface between the helical and GTPase domains and adjacent to the linker peptides. The reported ADH2‐causing Ser211Trp (S211W) mutation is located in the α2 helix of the GTPase domain;^6^ and the Val340Met (V340M) mutation reported by this study (shown in bold), and the previously reported Phe341Leu (F341L) mutation^3^ are located in the α5 helix of the GTPase domain. Coding‐regions are shaded gray and untranslated regions are represented by open boxes.

Autosomal dominant hypocalcemia (ADH) is a disorder of systemic calcium homeostasis that is associated with enhanced sensitivity of the calcium‐sensing receptor (CaSR) to extracellular calcium (Ca^2+^_o_) concentrations.^(1–3)^ The CaSR is a guanine‐nucleotide binding protein (G‐protein)‐coupled receptor (GPCR) that plays a pivotal role in Ca^2+^_o_ homeostasis by transducing increases in the prevailing Ca^2+^_o_ concentration into multiple signaling cascades that include G_q/11_‐protein–mediated activation of phospholipase C (PLC), which in the parathyroid glands and kidneys induces rapid increases in intracellular calcium (Ca^2+^_i_) that lead to decreased parathyroid hormone (PTH) secretion and increased urinary calcium excretion, respectively.^2^, ^4^, ^5^ ADH is a genetically heterogeneous disorder most commonly caused by germline gain‐of‐function mutations of the CaSR, which is encoded by the *CASR* gene on chromosome 3q21.1, and this is referred to as ADH type 1 (ADH1; OMIM #601198).^1^, ^2^, ^5^ However, some ADH patients and families have recently been shown to harbor germline mutations of G‐protein subunit‐α11 (Gα_11_), which is encoded by the *GNA11* gene (Fig. 1) on chromosome 19p13.3,^3^, ^6^, ^7^ and referred to as ADH type 2 (ADH2; OMIM #615361).^3^ These ADH‐associated Gα_11_ mutations have been demonstrated to enhance CaSR‐mediated signaling in cellular studies, consistent with a gain‐of‐function.^3^, ^7^ ADH1 patients have calcitropic phenotypes, such as hypocalcemia with inappropriately low or normal PTH concentrations and a relative hypercalciuria that is characterized by urinary calcium to creatinine ratios that are within or above the reference range,^1^, ^8^, ^9^ and mice with a gain‐of‐function CaSR mutation, that are representative of ADH1, have been reported to also have non‐calcitropic phenotypes such as cataracts.^10^ Although these features are similar to hypoparathyroidism, ADH1 is considered to represent a distinct disease entity from hypoparathyroidism, because affected individuals generally have PTH concentrations that are detectable and within the reference range.^1^, ^8^ Furthermore, ADH1 patients may also develop a Bartter‐like syndrome characterized by hypokalemic alkalosis, renal salt wasting, and hyperreninemic hyperaldosteronism,^11^, ^12^ and the use of active vitamin D metabolites to treat symptomatic ADH1 patients may result in the development of marked hypercalciuria, nephrocalcinosis, nephrolithiasis, and renal impairment.^1^, ^9^ In contrast to ADH1, the phenotypic spectrum of ADH2 has not been fully elucidated, especially because only five ADH2 probands with Gα_11_ mutations (Fig. 1) have been reported to date.^3^, ^6^, ^7^ Thus, it remains to be established whether ADH2 patients with germline gain‐of‐function Gα_11_ mutations are susceptible to hypercalciuria, particularly when treated with active vitamin D preparations, or at risk of a Bartter‐like syndrome. Moreover, Gα_11_ is a widely expressed protein that mediates the biological effects of GPCRs in a range of tissues,^13^ and it is currently unknown whether patients with ADH2 may harbor additional calcitropic and non‐calcitropic phenotypes. We ascertained a family with ADH and keratoconus, a non‐calcitropic disorder of the cornea, and hypothesized that either a single genetic abnormality may be causing ADH and keratoconus, or that ADH and keratoconus may be due to two different genetic abnormalities; ie, digenic inheritance. To explore these hypotheses, we undertook whole‐exome sequencing (WES) analysis of two relatives, both of whom had hypocalcemia, and one of whom also had keratoconus, to identify the causative variant(s).

## Patients and Methods

### Patients

**Figure 2:**
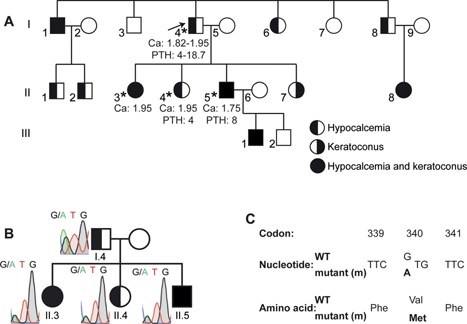
Identification of a Val340Met Gα_11_ mutation in a family with hypocalcemia and keratoconus. (*A*) Pedigree of the family, with males and females indicated by squares and circles, respectively. Individuals affected with hypocalcemia, keratoconus, or the combined occurrence of hypocalcemia and keratoconus are indicated by left half‐filled, right half‐filled, or filled symbols, respectively, and unaffected individuals are indicated by open symbols. The proband (individual I.4) is indicated by an arrow, and asterisks indicate those individuals from whom DNA was available for these studies. Plasma calcium (Ca) (mmol/L; normal range = 2.20 to 2.60 mmol/L) and plasma PTH (pg/mL; normal range = 12 to 65 pg/mL) values are shown for individuals I.4, II.3, II.4, and II.5. (*B*) A heterozygous G>A transition at nucleotide c.1018 within exon 7 of *GNA11* (Fig. 1) was identified by WES and confirmed by Sanger DNA sequence analysis. The c.1018G>A *GNA11* variant was detected in all four family members with hypocalcemia from whom samples were available. (*C*) This DNA sequence abnormality was predicted to lead to a missense amino acid substitution of Val to Met at codon 340.

The proband (individual I.4; Fig. 2 *A*) is a 66‐year‐old male, from Iran, who was diagnosed with hypocalcemia at the age of 16 years following a childhood history of intermittent muscle cramps. His serum adjusted‐calcium concentrations have ranged from 1.82 to 1.95 mmol/L (normal range = 2.20 to 2.60 mmol/L), in association with mild hyperphosphatemia (serum phosphate = 1.42 to 1.81 mmol/L; normal range = 0.80 to 1.45 mmol/L), normal serum creatinine concentrations of 72 to 80 µmol/L (normal range = 44 to 133 µmol/L), low/normal serum PTH concentrations of 4.0 to 18.7 pg/mL (normal range = 12 to 65 pg/mL), a normal 25‐hydroxyvitamin D concentration of 18.7 ng/mL (normal range = 10 to 50 ng/mL), and a normal 1,25‐dihydroxyvitamin D concentration of 25.7 pg/mL (normal range = 16 to 45 pg/mL). He had been treated with oral calcium and active vitamin D preparations, and developed non‐obstructive bilateral nephrolithiasis in association with urinary calcium values ranging from 4.3 to 10.2 mmol/24 hours (normal range = 2.5 to 7.5 mmol/24 hours). He was found to have mild bilateral cataracts at the age of 62 years. His family history revealed that 11 relatives (7 males and 4 females) over three generations had hypocalcemia, with symptoms such as seizures, muscle cramps, and tetany. Affected relatives also had low circulating PTH concentrations, and relative or absolute hypercalciuria (Fig. 2 *A*), and these findings supported a diagnosis of ADH. Five hypocalcemic family members (3 males and 2 females) and one normocalcemic family member also had keratoconus, a corneal disorder, which in individuals II.3, II.5, and II.7 (Fig. 2 *A*) had developed between the ages of 30 and 34 years.^14^ There was no consanguinity and the inheritance of hypocalcemia from father to son in individuals; eg, from I.1 to II.1 and II.2; I.4 to II.5; and II.5 to III.1 (Fig. 2 *A*), indicated that hypocalcemia was transmitted as an autosomal dominant disorder. Similarly, the inheritance of keratoconus from father to son in individuals II.5 to III.1 (Fig. 2 *A*), may be because keratoconus is transmitted as an autosomal dominant disorder, but the occurrence of keratoconus in the children (II.3, II.5 and II.7; and II.8) of unaffected parents (I.4 and I.5; and I.8 and I.9, respectively, Fig. 2 *A*) indicates an autosomal recessive inheritance or autosomal dominant inheritance with reduced penetrance. To investigate the cause of the hypocalcemia and keratoconus in this family, venous blood samples were collected from four available patients, of whom two had hypocalcemia only (individuals I.4 and II.4), and two others had both hypocalcemia and keratoconus (individuals II.3 and II.5; Fig. 2 *A*). Leukocyte DNA was extracted using the Gentra PureGene blood kit (Qiagen, Crawley, UK). CaSR mutations, which would cause ADH1, and mutations of three genes, *GCMB*, *PTH*, and *AIRE1*, which are known to cause hypoparathyroidism had been previously excluded by an accredited genetics diagnostic laboratory. Informed consent was obtained from all participants included in the study, using protocols approved by the UK Multicentre Research Ethics Committee (MREC/02/2/93) and the Hospital Research Ethics in Israel.

### Exome capture and DNA sequence analysis

Exome capture was performed in individual I.4, who was affected with hypocalcemia, and in individual II.5, who was affected with both hypocalcemia and keratoconus (Fig. 2 *A*), using the SeqCap EZ Human Exome Library v2.0 (Roche NimbleGen, Madison, WI, USA),^15^ and DNA sequences determined using a 100‐bp paired‐end read protocol on an Illumina HiSeq2000 platform.^15^ A minimum of 20× vertical read depth was obtained for >88% of the coding exome, as specified by the consensus coding sequence (CCDS) project,^16^ in both individuals. Reads were aligned to the Human Sequence version 37d5 (hs37d5) reference genome using Stampy^17^ and variant calling of single nucleotide variants (SNVs) and short insertions and deletions (indels) was undertaken using Platypus (v0.5.1).^18^ Analysis of coding variants was undertaken using Ingenuity Variant Analysis. To search for variants causing hypocalcemia and keratoconus under autosomal dominant inheritance models, variants with a minor allele frequency (MAF) >5% within all populations of 100 genomes data (April 2012 phase 1 call set [v3 update]) and from the National Heart, Lung, and Blood Institute (NHLBI) exome sequencing project (Exome Variant Server, NHLBI GO Exome Sequencing Project (ESP), Seattle, WA (http://evs.gs.washington.edu/EVS/), were excluded. To search for variants causing keratoconus under an autosomal recessive inheritance model, variants with a MAF >5% were not excluded. The pathogenicity of variants was inferred from several criteria: allele frequency within the NHLBI exome sequencing project and the Exome Aggregation Consortium (ExAC) (Cambridge, MA, USA; http://exac.broadinstitute.org/), amino acid conservation, physicochemical alterations in amino acid substitutions, splice site prediction algorithms (NNSPLICE [http://www.fruitfly.org/seq_tools/splice.html], MaxEntScan [http://genes.mit.edu/burgelab/maxent/Xmaxentscan_scoreseq.html], GeneSplicer [http://www.cbcb.umd.edu/software/GeneSplicer/gene_spl.shtml], and SpliceSiteFinder‐Like [http://www.interactive-biosoftware.com]), and literature review. Variants of interest were confirmed by polymerase chain reaction (PCR) and Sanger DNA sequence analysis of the appropriate exons, using leukocyte DNA samples from the four available family members and two unrelated unaffected individuals, as reported.^19^

### Protein sequence alignments and three‐dimensional modeling

Amino acid sequences of Gα‐subunit paralogs and transforming growth factor, beta‐induced (TGFBI) orthologs were aligned using the Clustal Omega program (European Bioinformatics Institute, the European Molecular Biology Laboratory). The crystal structure of Gα_q_ in complex with the GTP analogue guanosine diphosphate (GDP)‐AIF_4_ has been determined (Protein Data Bank [PDB] accession number 3OHM),^20^ and this was used to model Gα_11_, which has 90% identity with Gα_q_ at the amino acid level, using the PyMOL Molecular Graphics System (version 1.2r3pre; Schrodinger, LLC).

### Cell culture and transfection

The generation of wild‐type and mutant Leu341 pBI‐CMV2‐*GNA11* expression constructs have been reported.^3^ Site‐directed mutagenesis was used to generate the Val to Met mutation at codon 340 in the pBI‐CMV2‐*GNA11* construct using the Quikchange Lightning Site‐directed Mutagenesis kit (Agilent Technologies, Santa Clara, CA, USA) and gene‐specific primers (SigmaAldrich, St Louis, MO, USA), as reported.^19^ For all studies, empty vector, wild‐type, or mutant pBI‐CMV2‐*GNA11* expression constructs were transiently transfected into human embryonic kidney (HEK) 293 cells stably expressing the CaSR (HEK293‐CaSR) using Lipofectamine 2000 (LifeTechnologies, Carlsbad, CA, USA).^21^ Cells were maintained in DMEM‐Glutamax media (ThermoFisher, Waltham, MA, USA) with 10% fetal bovine serum (Gibco, Carlsbad, CA, USA) and 400 μg/mL geneticin (ThermoFisher) at 37°C, 5% CO_2_. Successful transfection was confirmed by visualizing GFP fluorescence using an Eclipse E400 fluorescence microscope with a Y‐FL Epifluorescence attachment and a triband 4,6‐diamidino‐2‐phenylindole‐FITC‐Rhodamine filter, and images captured using a DXM1200C digital camera and NIS Elements software (Nikon).^3^, ^21^, ^22^ The expression of Gα_11_, GFP, calnexin, and CaSR proteins was also determined by Western blot analysis using anti‐Gα_11_ (Santa Cruz Biotechnology, Dallas, TX, USA), anti‐GFP (Santa Cruz), anti‐calnexin (Millipore, Billerica, MA, USA), or anti‐CaSR (Abcam, Cambridge, UK) antibodies. The Western blots were visualized using Immuno‐Star WesternC kit (BioRad, Hercules, CA, USA) on a BioRad Chemidoc XRS+ system.^3^

### Intracellular calcium measurements

The Ca^2+^_i_ responses of HEK293‐CaSR cells expressing wild‐type or mutant Gα_11_ proteins were assessed by a flow cytometry–based assay, as reported.^2^, ^3^, ^21^, ^22^ In brief, HEK293‐CaSR cells were plated in T75 flasks and transiently transfected 24 hours later with 16 μg DNA.^3^ At 48 hours posttransfection, cells were detached, resuspended in calcium (Ca^2+^)‐free and magnesium (Mg^2+^)‐free Hanks buffered saline solution (HBSS) and loaded with 1 μg/mL Indo‐1‐acetoxymethylester (Indo‐1‐AM) for 1 hour at 37°C. After removal of free dye, the cells were resuspended in Ca^2+^‐free and Mg^2+^‐free HBSS and maintained at 37°C. Transfected cells, in suspension, were analyzed by flow cytometry on a MoFlo modular flow cytometer (Beckman Coulter, Indianapolis, IN, USA) and data on the Ca^2+^_i_ responses to alterations in Ca^2+^_o_ collected from all cells that expressed GFP, by simultaneous measurements of GFP expression (at 525 nm), Ca^2+^_i_‐bound Indo‐1AM (at 410 nm), and free Indo‐1AM (ie, not bound to Ca^2+^_i_) (at 485 nm), using a JDSU Xcyte laser (Coherent Radiation, Santa Clara, CA, USA), on each cell at each Ca^2+^_o_ concentration [Ca^2+^]_o_, as described.^3^, ^21^ Assays were performed in four biological replicates for each of the expression constructs. The peak mean fluorescence ratio of the transient response after each individual stimulus was measured using Cytomation Summit software (Beckman Coulter), and expressed as a normalized response, as described.^3^, ^21^ Nonlinear regression of concentration‐response curves was performed with GraphPad Prism using the normalized response at each [Ca^2+^]_o_ for each separate experiment for the determination of the EC_50_ (ie, [Ca^2+^]_o_ required for 50% of the maximal response). Data is presented as mean with confidence interval or mean ± standard error of the mean (SE). Statistical analysis was performed using the F‐test. A value of *p* < 0.05 was considered significant for all analyses.

## Results

### Identification of a novel Val340Met Gα_11_ mutation

**Figure 3:**
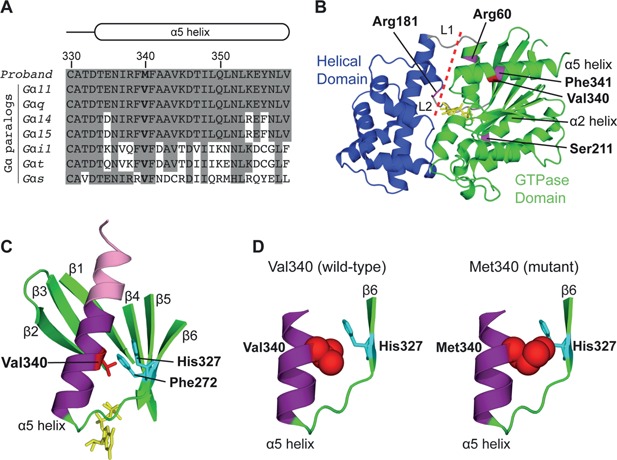
Predicted effects of the Val340Met mutation on the Gα_11_ protein. (*A*) Multiple protein sequence alignment of residues comprising the α5 helix of Gα‐subunit paralogs. The wild‐type Val340 (V) and mutant Met340 (M) residues are shown in bold. Conserved residues are shaded gray. (*B*) Overall three‐dimensional structure of the Gα_11_ protein. The Gα‐subunit helical (blue) and GTPase (green) domains are connected by the L1 and L2 peptides (gray). The four Gα_11_ residues, which have been previously reported to be mutated in ADH2,^3^, ^6^, ^7^ are shown in magenta. The mutated Arg60 and Arg181 residues are located at the interdomain interface (red dashed line), which forms a pocket for the binding of guanine nucleotides (yellow). The GTPase domain is the location of the mutated Ser211 residue (α2 helix), and mutated Val340 (red) and Phe341 residues (α5 helix). (*C*) Close‐up view of the α5 helix, which comprises a C‐terminal interface module (pink) that interacts with partner GPCRs and an N‐terminal transmission module (purple), which interacts with the surrounding β1‐β6 strands to facilitate conformational changes that lead to guanine nucleotide exchange and Gα‐subunit activation.^23^ The wild‐type Val340 residue (red) is predicted to form non‐polar interactions with the Phe272 and His327 residues (cyan), 26 which are located in the β5 and β6 strands, respectively. (*D*) The introduction of a mutant Met340 residue (red, space filling model) is predicted to sterically hinder His327 (cyan, stick model), thereby altering the conformation of the α5 helix and/or β6 strand.

Exome capture and high‐throughput sequence analysis of genomic DNA from two affected individuals, one with hypocalcemia (individual I.4) and the other with hypocalcemia and keratoconus (individual II.5) (Fig. 2 *A*) resulted in the detection of >5000 non‐dbSNP variants in both individuals. The exclusion of variants with a MAF of >5% resulted in the identification of 104 novel variants in 103 genes, of which three variants were excluded as sequence artifacts as they occurred within trinucleotide repeats. The only variant identified in a gene previously associated with hypocalcemia, was a heterozygous G>A transition at nucleotide c.1018 (c.1018G>A), located in exon 7 of the *GNA11* gene (Fig. 1). The variant was confirmed by Sanger DNA sequencing to be present in both patients affected with hypocalcemia (individuals I.4 and II.5) and in two other family members (individuals II.3 and II.4) with hypocalcemia from whom samples were available (Fig. 2 *B*). This G>A transition (GTG to ATG) resulted in a missense substitution, Val340Met, of the encoded Gα_11_ protein (Fig. 2 *C*). The absence of this DNA sequence abnormality in >60,000 exomes from the Exome Aggregation Consortium (ExAC), together with evolutionary conservation of the Val340 residue in vertebrate Gα‐subunit paralogs (Fig. 3 *A*) indicated that the Val340Met abnormality likely represented a pathogenic *GNA11* mutation rather than a benign polymorphic variant.

### Identification of candidate variant for keratoconus

Whole‐exome sequencing revealed a novel heterozygous c.1209T>G transversion in *TGFBI*, encoding a variant His403Gln in TGFBI, which was present in both individuals (I.4 and II.5, Supporting Fig. 1 *A*, Supporting Table  1). The TGFBI His403Gln variant is not present in ExAC, although a different variant in the same amino acid, His403Tyr, is present in only 3 out of >60,000 individuals. The His403 residue is also evolutionarily conserved in TGFBI orthologs (Supporting Fig. 1 *B*). The *TGFBI* variant was confirmed by Sanger DNA sequence analysis in the siblings, II.3 and II.5, who had hypocalcemia and keratoconus, but its absence in the sister II.4, who has hypocalcemia only, indicates that this TGFBI variant is not involved in the etiology of hypocalcemia (Supporting Fig. 1 *A*). Moreover, the presence of this TGFBI variant in the father (individual I.4), who was affected with hypocalcemia only, indicates likely nonpenetrance of the mutant allele (Supporting Fig.  1*A*).

### Structural characterization of the Val340Met Gα_11_ mutant protein

To evaluate whether the Val340Met *GNA11* variant represents a pathogenic mutation and the cause of hypocalcemia in this family, structural and functional studies were undertaken. The crystal structure of Gα_q_, which shares 90% amino acid identity with Gα_11_,^3^, ^20^ was used to predict the effects of the Val340Met mutation. The mutated Val340 residue is located within the α5 helix of the GTPase domain of Gα_11_ (Fig. 3 *B*, *C*), and next to the Phe341 residue, which has been reported to be associated with a gain‐of‐function Phe341Leu mutation (Figs. 1 and 3*B*) causing ADH2.^3^ The α5 helix is located at the C‐terminus of Gα‐subunits and plays a critical role in GPCR binding and G‐protein activation.^23^, ^24^ Indeed, the C‐terminal portion of the α5 helix, which is known as the interface module, directly binds to the transmembrane domain and intracellular loops of activated GPCRs,^24^ whereas the N‐terminal portion, which is known as the transmission module, interacts with the GTPase domain β‐sheet, comprising the β1‐β6 strands (Fig. 3 *C*), to mediate conformational changes in Gα‐subunit structure that facilitate guanine nucleotide exchange.^23^, ^25^ The Val340 residue is predicted to form non‐polar interactions with the conserved Phe272 and His327 residues, located in the β5 and β6 strands, respectively (Fig. 3 *C*), which are considered to stabilize the binding of G‐protein with ligand‐bound GPCR.^23^, ^25^, ^26^ The Val340Met mutation is predicted to impair the interaction with the His327 Gα_11_ residue (Fig. 3 *D*), thereby altering the conformation of the α5 helix and/or β6 strand, and destabilizing the Gα‐GPCR complex.

### Functional characterization of the Val340Met Gα_11_ mutant protein

**Figure 4:**
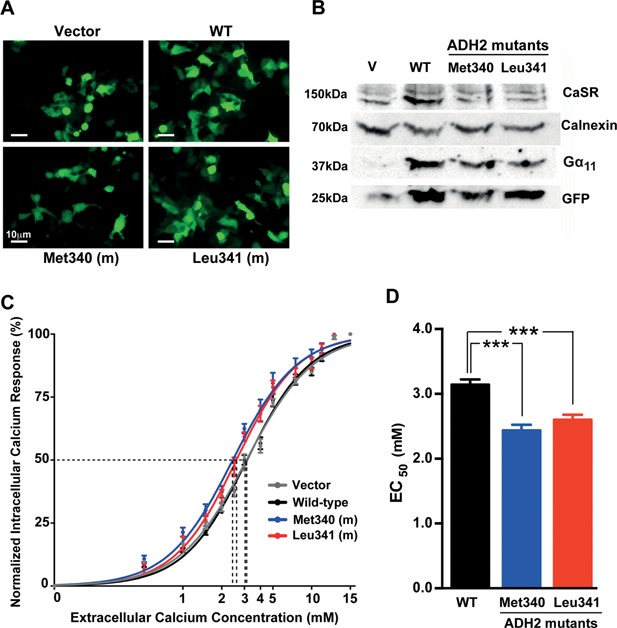
Functional characterization of wild‐type and ADH2‐associated mutant Gα_11_ proteins. (*A*) Fluorescence microscopy of HEK293 cells stably expressing CaSR (HEK293‐CaSR) and transiently transfected with wild‐type (WT) or ADH2‐associated mutant (m) (Met340 and Leu341) pBI‐CMV2‐*GNA11* constructs, or with vector (V) only. Detection of GFP in these cells indicates successful transfection and expression of these constructs. Bar indicates 10 µm. (*B*) Western blot analysis of whole‐cell lysates using antibodies to CaSR, calnexin, Gα_11_, and GFP. Transient transfection of WT or ADH2‐associated mutant constructs resulted in overexpression of Gα_11_ when normalized to calnexin expression. (*C*) Concentration‐response curves showing normalized Ca^2+^_i_ response to changes in [Ca^2+^]_o_ of HEK293‐CaSR cells transfected with WT or ADH2‐associated Gα_11_ mutants. The Ca^2+^_i_ responses are shown as the mean ± SEM of 4 independent transfections. The ADH2‐associated Gα_11_ mutants (Met340 and Leu341) led to a leftward shift of the concentration‐response curves (blue and red, respectively) when compared with WT Gα_11_ (black), which harbors Val and Phe residues at codons 340 and 341, respectively. (*D*) The Met340 and Leu341 mutants (blue and red bars, respectively) were associated with significantly decreased EC_50_ values compared to cells expressing WT Gα_11_ (black bar). ***p < 0.0001.

To determine the effects of these predicted changes in Gα_11_ structure (Fig. 3) on CaSR‐mediated signaling, bidirectional pBI‐CMV2‐*GNA11* expression constructs containing wild‐type Val340, mutant Met340, or the previously reported gain‐of‐function Leu341 mutant *GNA11* cDNA,^3^ or vector containing the GFP reporter gene alone were transiently transfected into HEK293 cells stably expressing the CaSR. Expression of CaSR, Gα_11_, and GFP was confirmed by fluorescence microscopy and/or Western blot analysis (Fig. 4 *A*, *B*). Calnexin was used as a loading control in Western blot analyses, and Gα_11_ expression was demonstrated to be similar in cells transiently transfected with wild‐type or mutant Gα_11_ proteins, and greater than that of cells transfected with the empty pBI‐CMV2 vector (Fig. 4 *B*). The responses of [Ca^2+^]_i_ to alterations in [Ca^2+^]_o_ in cells expressing the different *GNA11* vectors were assessed by flow cytometry. The Ca^2+^_i_ responses in wild‐type and mutant Gα_11_‐expressing cells were shown to increase in a dose‐dependent manner following stimulation with increasing concentrations of Ca^2+^_o_ between 0 and 15 mM. However, expression of the Met340 and Leu341 mutant Gα_11_ proteins resulted in a leftward shift of the concentration‐response curves (Fig. 4 *C*), with significantly lower half maximal (EC_50_) values, compared to wild‐type Val340 Gα_11_ and empty vector (Met340 = 2.44 mM [95% CI, 2.31 to 2.77 mM]; Val340 = 3.14 mM [95% CI. 3.03 to 3.26 mM]; empty vector = 3.12 mM [95% CI, 2.99 to 3.25 mM]; *p* < 0.0001) (Fig. 4 *D*). There was no significant difference in the EC_50_ values between Met340 and Leu341 (Leu341 = 2.60 mM; 95% CI, 2.49 to 2.72 mM; *p* > 0.05). Thus, the Val340Met missense substitution represents a novel gain‐of‐function Gα_11_ mutation, similar to those reported in ADH2 patients.^3^, ^7^

## Discussion

The identification of a germline gain‐of‐function Gα_11_ mutation, Val340Met, indicates ADH2 to be the likely cause of hypocalcemia in this family. To date, five other hypocalcemic patients and families have been reported to harbor germline Gα_11_ mutations, consistent with a diagnosis of ADH2.^3^, ^6^, ^7^ Similar to the family characterized in this study, individuals with germline gain‐of‐function Gα_11_ mutations have been reported to generally have mild‐to‐moderate hypocalcemia (serum adjusted‐calcium concentrations ranging from 1.75 to 2.15 mmol/L) in association with serum PTH concentrations that are detectable and within the lower half of the normal range.^3^, ^6^, ^7^ However, despite ADH2 being associated with mild biochemical features, affected individuals are commonly symptomatic and may present with paresthesia, muscle cramps, carpo‐pedal spasms, or seizures.^3^, ^6^, ^7^ Moreover, as described for the proband in this study (individual I.4, Fig. 2 *A*), some ADH2 patients appear susceptible to treatment‐related hypercalciuria, nephrocalcinosis, and nephrolithiasis,^7^ although affected individuals likely have a milder urinary phenotype, with significantly reduced urinary calcium excretion compared to ADH1 patients who harbor gain‐of‐function CaSR mutations.^7^ Furthermore, patients with germline heterozygous gain‐of‐function Gα_11_ mutations, in contrast to patients with germline heterozygous gain‐of‐function CaSR mutations, may have non‐calcitropic phenotypes. For example, short stature caused by postnatal growth insufficiency was reported in affected individuals from a multigenerational kindred with hypocalcemia and a germline heterozygous gain‐of‐function mutation, Arg60Leu,^7^ and it is possible that the occurrence of mild bilateral cataracts in the proband (individual I.4) may also be due to the germline gain‐of‐function Gα_11_ mutation, Val340Met. It is also of interest to note that somatic Gα_11_ mutations that lead to constitutive G‐protein activation are associated with the development of uveal melanomas.^27^ However, keratoconus, a corneal disorder, was not observed in all patients harboring the germline Val340Met Gα_11_ mutation, thereby indicating that keratoconus is not due to the Gα_11_ mutation. Instead, keratoconus in this family is likely due to the identified novel germline variant, His403Gln in TGFBI (Supporting Fig. 1), abnormalities of which have previously been reported in association with keratoconus and autosomal dominant forms of corneal dystrophy.^28^, ^29^ Thus, the occurrence of keratoconus and ADH2 in this family are not genetically associated, but are instead due to digenic inheritance of mutations involving TGFBI and Gα_11_.

Structural and functional studies of ADH2‐causing gain‐of‐function Gα_11_ mutations have revealed residues and peptide motifs critical for Gα‐subunit function. Thus, the importance of the interface at which the Gα‐subunit helical and GTPase domains interact to mediate guanine nucleotide exchange, has been demonstrated by the reported Arg60Leu, Arg60Cys, and Arg181Gln Gα_11_ mutations,^3^, ^6^, ^7^ which are located at the interdomain interface (Fig. 3 *B*), 25 and predicted to disrupt GDP binding.^3^, ^6^, ^7^ Whereas, the reported ADH2‐causing Ser211Trp mutation, located in the α2 helix of the GTPase domain (Fig. 3 *B*), may cause a gain‐of‐function by dissociating the Gα‐subunit from the Gβγ heterodimer.^6^ Furthermore, the reported ADH2‐causing Phe341Leu mutation^3^ is located in the C‐terminal α5 helix, which facilitates G‐protein‐GPCR coupling,^23^ and the mutated Phe341 residue is predicted to play major roles in G‐protein activation and inactivation.^25^, ^26^ Indeed, under basal conditions, Phe341 forms part of a hydrophobic cluster of phenylalanine residues that maintains the Gα‐subunit in an inactive GDP‐bound conformation.^25^, ^26^ Upon ligand‐binding, Phe341 binds to the second intracellular loop of the activated cognate GPCR,^24^ which disrupts the hydrophobic cluster, leading to the release of GDP.^25^, ^26^ The Phe341Leu mutation is predicted to affect the hydrophobic cluster,^3^ thereby leading to GDP/GTP exchange and G‐protein activation. In contrast to these roles of Phe341, the neighboring Val340 residue does not bind to activated GPCRs and is not involved in GDP/GTP exchange,^24^, ^25^ but instead may play a role in stabilizing the nucleotide‐free conformation of the Gα‐subunit once bound to activated GPCR.^23^ Thus, the Val340Met mutation likely mediates Gα_11_ activation by influencing the stability of Gα‐GPCR interactions. These different ADH2 mutations have yielded insights into Gα‐subunit structure‐function relationships, which may aid the design of novel targeted therapeutic agents for the treatment of ADH, for which there is currently a lack of adequate drugs.^30^

In conclusion, we have identified a novel germline gain‐of‐function Gα_11_ mutation, Val340Met, that causes ADH2 in a family in which some members also suffered from keratoconus. Keratoconus in this family was likely due to a novel germline TGFBI His403Gln variant, thereby indicating that keratoconus and ADH2 are not caused by the same mutation, but instead the combined occurrence of ADH2 and keratoconus in some family members is due to digenic inheritance. Finally, our study provides an exemplar of the clinical utility of WES, that enabled the identification of two likely genetic causes by a single experimental protocol, which would not have been readily possible using former genetic techniques.

## Disclosures

All authors state that they have no conflicts of interest.

## Supporting information

Supporting Fig 1 Legend.

Supporting Fig S1.

Supporting Table S1.

## References

[1] Pearce SH , Williamson C , Kifor O , et al. A familial syndrome of hypocalcemia with hypercalciuria due to mutations in the calcium‐sensing receptor. N Engl J Med. 1996; 335(15):1115–22. 8813042 10.1056/NEJM199610103351505

[2] Hannan FM , Nesbit MA , Zhang C , et al. Identification of 70 calcium‐sensing receptor mutations in hyper‐ and hypo‐calcaemic patients: evidence for clustering of extracellular domain mutations at calcium‐binding sites. Hum Mol Genet. 2012; 21(12):2768–78. 22422767 10.1093/hmg/dds105

[3] Nesbit MA , Hannan FM , Howles SA , et al. Mutations affecting G‐protein subunit alpha11 in hypercalcemia and hypocalcemia. N Engl J Med. 2013; 368(26):2476–86. 23802516 10.1056/NEJMoa1300253 PMC3773604

[4] Hofer AM , Brown EM. Extracellular calcium sensing and signalling. Nat Rev Mol Cell Biol. 2003; 4(7):530–8. 12838336 10.1038/nrm1154

[5] Hannan FM , Thakker RV. Calcium‐sensing receptor (CaSR) mutations and disorders of calcium, electrolyte and water metabolism. Best Pract Res Clin Endocrinol Metab. 2013; 27(3):359–71. 23856265 10.1016/j.beem.2013.04.007

[6] Mannstadt M , Harris M , Bravenboer B , et al. Germline mutations affecting Galpha11 in hypoparathyroidism. N Engl J Med. 2013; 368(26):2532–4. 23802536 10.1056/NEJMc1300278 PMC3750735

[7] Li D , Opas EE , Tuluc F , et al. Autosomal dominant hypoparathyroidism caused by germline mutation in GNA11: phenotypic and molecular characterization. J Clin Endocrinol Metab. 2014; 99(9):E1774–83. 24823460 10.1210/jc.2014-1029 PMC4154081

[8] Raue F , Pichl J , Dorr HG , et al. Activating mutations in the calcium‐sensing receptor: genetic and clinical spectrum in 25 patients with autosomal dominant hypocalcaemia − a German survey. Clin Endocrinol (Oxf). 2011; 75(6):760–5. 21645025 10.1111/j.1365-2265.2011.04142.x

[9] Yamamoto M , Akatsu T , Nagase T , Ogata E. Comparison of hypocalcemic hypercalciuria between patients with idiopathic hypoparathyroidism and those with gain‐of‐function mutations in the calcium‐sensing receptor: is it possible to differentiate the two disorders? J Clin Endocrinol Metab. 2000; 85(12):4583–91. 11134112 10.1210/jcem.85.12.7035

[10] Hough TA , Bogani D , Cheeseman MT , et al. Activating calcium‐sensing receptor mutation in the mouse is associated with cataracts and ectopic calcification. Proc Natl Acad Sci U S A. 2004; 101(37):13566–71. 15347804 10.1073/pnas.0405516101 PMC518795

[11] Vargas‐Poussou R , Huang C , Hulin P , et al. Functional characterization of a calcium‐sensing receptor mutation in severe autosomal dominant hypocalcemia with a Bartter‐like syndrome. J Am Soc Nephrol. 2002; 13(9):2259–66. 12191970 10.1097/01.asn.0000025781.16723.68

[12] Watanabe S , Fukumoto S , Chang H , et al. Association between activating mutations of calcium‐sensing receptor and Bartter's syndrome. Lancet. 2002; 360(9334):692–4. 12241879 10.1016/S0140-6736(02)09842-2

[13] Wettschureck N , Offermanns S. Mammalian G proteins and their cell type specific functions. Physiol Rev. 2005; 85(4):1159–204. 16183910 10.1152/physrev.00003.2005

[14] Vazirani J , Basu S. Keratoconus: current perspectives. Clin Ophthalmol. 2013; 7:2019–30. 24143069 10.2147/OPTH.S50119 PMC3798205

[15] Taylor JC , Martin HC , Lise S , et al. Factors influencing success of clinical genome sequencing across a broad spectrum of disorders. Nat Genet. 2015; 47(7):717–26. 25985138 10.1038/ng.3304 PMC4601524

[16] Pruitt KD , Harrow J , Harte RA , et al. The consensus coding sequence (CCDS) project: identifying a common protein‐coding gene set for the human and mouse genomes. Genome Res. 2009; 19(7):1316–23. 19498102 10.1101/gr.080531.108 PMC2704439

[17] Lunter G , Goodson M. Stampy: a statistical algorithm for sensitive and fast mapping of Illumina sequence reads. Genome Res. 2011; 21(6):936–9. 20980556 10.1101/gr.111120.110 PMC3106326

[18] Rimmer A , Phan H , Mathieson I , et al. Integrating mapping‐, assembly‐ and haplotype‐based approaches for calling variants in clinical sequencing applications. Nat Genet. 2014; 46(8):912–8. 25017105 10.1038/ng.3036 PMC4753679

[19] Newey PJ , Gorvin CM , Cleland SJ , et al. Mutant prolactin receptor and familial hyperprolactinemia. N Engl J Med. 2013; 369(21):2012–20. 24195502 10.1056/NEJMoa1307557 PMC4209110

[20] Waldo GL , Ricks TK , Hicks SN , et al. Kinetic scaffolding mediated by a phospholipase C‐beta and Gq signaling complex. Science. 2010; 330(6006):974–80. 20966218 10.1126/science.1193438 PMC3046049

[21] Nesbit MA , Hannan FM , Howles SA , et al. Mutations in AP2S1 cause familial hypocalciuric hypercalcemia type 3. Nat Genet. 2013; 45(1):93–7. 23222959 10.1038/ng.2492 PMC3605788

[22] Hannan FM , Howles SA , Rogers A , et al. Adaptor protein‐2 sigma subunit mutations causing familial hypocalciuric hypercalcaemia type 3 (FHH3) demonstrate genotype‐phenotype correlations, codon bias and dominant‐negative effects. Hum Mol Genet. 2015; 24(18):5079–92. 26082470 10.1093/hmg/ddv226 PMC4550820

[23] Flock T , Ravarani CN , Sun D , et al. Universal allosteric mechanism for Galpha activation by GPCRs. Nature. 2015; 524(7564):173–9. 26147082 10.1038/nature14663 PMC4866443

[24] Rasmussen SG , DeVree BT , Zou Y , et al. Crystal structure of the beta2 adrenergic receptor‐Gs protein complex. Nature. 2011; 477(7366):549–55. 21772288 10.1038/nature10361 PMC3184188

[25] Sun D , Flock T , Deupi X , et al. Probing Galphai1 protein activation at single‐amino acid resolution. Nat Struct Mol Biol. 2015; 22(9):686–94. 26258638 10.1038/nsmb.3070 PMC4876908

[26] Kaya AI , Lokits AD , Gilbert JA , Iverson TM , Meiler J , Hamm HE A conserved phenylalanine as a relay between the alpha5 helix and the GDP binding region of heterotrimeric Gi protein alpha subunit. J Biol Chem. 2014; 289(35):24475–87. 25037222 10.1074/jbc.M114.572875 PMC4148873

[27] Van Raamsdonk CD , Griewank KG , Crosby MB , et al. Mutations in GNA11 in uveal melanoma. N Engl J Med. 2010; 363(23):2191–9. 21083380 10.1056/NEJMoa1000584 PMC3107972

[28] Guan T , Liu C , Ma Z , Ding S. The point mutation and polymorphism in keratoconus candidate gene TGFBI in Chinese population. Gene. 2012; 503(1):137–9. 22575726 10.1016/j.gene.2012.04.061

[29] Stewart HS , Ridgway AE , Dixon MJ , Bonshek R , Parveen R , Black G. Heterogeneity in granular corneal dystrophy: identification of three causative mutations in the TGFBI (BIGH3) gene‐lessons for corneal amyloidogenesis. Hum Mutat. 1999; 14(2):126–32. 10425035 10.1002/(SICI)1098-1004(1999)14:2<126::AID-HUMU4>3.0.CO;2-W

[30] Hannan FM , Walls GV , Babinsky VN , et al. The calcilytic agent NPS 2143 rectifies hypocalcemia in a mouse model with an activating calcium‐sensing receptor (CaSR) mutation: relevance to autosomal dominant hypocalcemia type 1 (ADH1). Endocrinology. 2015; 156(9):3114–21. 26052899 10.1210/en.2015-1269 PMC4541614

