# Supplementary material for: Identification of a G‐Protein Subunit‐α11 Gain‐of‐Function Mutation, Val340Met, in a Family With Autosomal Dominant Hypocalcemia Type 2 (ADH2)
Source: J Bone Miner Res. 2016 Jun 2;31(6):1207–14. doi: 10.1002/jbmr.2797 (PMC4915495; doi:10.1002/jbmr.2797)
Supplement: Supplementary file 1 — Supporting Fig 1 Legend. [file JBMR-31-1207-s001.doc]

**Supporting Fig. 1.** Identification of a novel *TGFBI* variant. *(A)* A novel T>G transversion in *TGFBI* identified by WES was confirmed by Sanger DNA sequencing and found to be present in the father (individual I.4, proband, indicated by arrow) without keratoconus and a son and daughter with keratoconus, but absent in a daughter without keratoconus. Males and females are indicated by squares and circles, respectively; individuals affected with hypocalcemia, keratoconus or the combined occurrence of hypocalcemia and keratoconus are indicated by left half-filled, right half-filled or filled symbols, respectively, and unaffected individuals are indicated by open symbols. *(B)* Multiple sequence alignment of TGFBI orthologs. Conserved residues are shaded in gray. The His403 (H403) residue (bold, arrowed) is conserved in TGFBI orthologs.
