# Supplementary figures and images for: Identification of a G‐Protein Subunit‐α11 Gain‐of‐Function Mutation, Val340Met, in a Family With Autosomal Dominant Hypocalcemia Type 2 (ADH2)
Source: J Bone Miner Res. 2016 Jun 2;31(6):1207–14. doi: 10.1002/jbmr.2797 (PMC4915495; doi:10.1002/jbmr.2797)

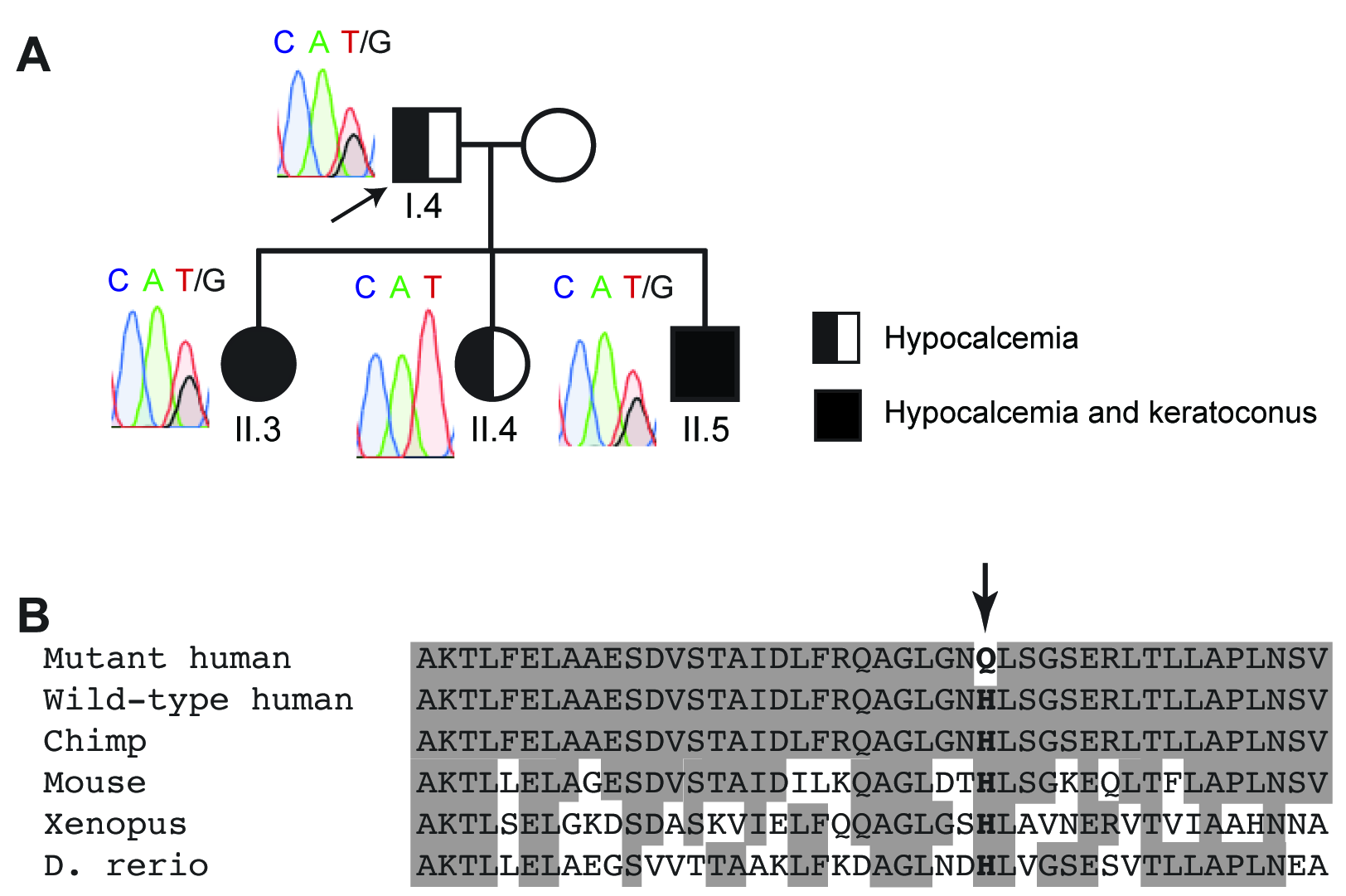

Supplement: Supplementary file 2 — Supporting Fig S1. [file JBMR-31-1207-s002.tif]
