# Supplementary material for: Identification of a G‐Protein Subunit‐α11 Gain‐of‐Function Mutation, Val340Met, in a Family With Autosomal Dominant Hypocalcemia Type 2 (ADH2)
Source: J Bone Miner Res. 2016 Jun 2;31(6):1207–14. doi: 10.1002/jbmr.2797 (PMC4915495; doi:10.1002/jbmr.2797)
Supplement: Supplementary file 3 — Supporting Table S1. [file JBMR-31-1207-s003.docx]

**Supporting Table 1.** Genetic causes of keratoconus and corneal dystrophies

| Disease/Locus | Chromosome | Gene | Status | Inheritance |
| --- | --- | --- | --- | --- |
| Keratoconus locus 1 (*KTCN1*) | 20p11.21 | Visual System Homeobox 1 (*VSX1*) | Candidate | Dominant or Recessive |
| Keratoconus locus 7 (*KTCN7*) | 13q32 | Dedicator of Cytokinesis 9 (*DOCK9*) | Candidate | Not known |
| Brittle cornea syndrome 1 | 16q24.2 | Zinc finger protein 469 (*ZNF469*) | Established | Recessive |
| Brittle cornea syndrome 2 | 4q27 | PR domain-containing protein 5 (*PRDM5*) | Established | Recessive |
| Lattice corneal dystrophy type 1 | 5q31.1 | Transforming growth factor beta-induced (*TGFBI*) | Established | Dominant |
| Leber congenital amaurosis (LCA)1 | 17p13.1 | Retinal guanylate cyclase (*GUCY2D*) | Established | Recessive |
| LCA2 | 1p31.3-p31.2 | Retinal pigment epithelium specific protein, 65kD (*RPE65*) | Established | Recessive |
| LCA3 | 14q31.3 | Spermatogenesis-associated protein 7 (*SPATA7*) | Established | Recessive |
| LCA4 | 17p13.2 | Arylhydrocarbon-interacting protein-like-1 (*AIPL1*) | Established | Recessive |
| LCA5 | 6q14.1 | Lebercilin (*LCA5*) | Established | Recessive |
| LCA6 | 14q11.2 | Retinitis pigmentosa GTPase regulator-interacting protein (*RPGRIP1*) | Established | Recessive |
| LCA7 | 19q13.33 | Cone-rod homeobox-containing gene (*CRX*) | Established | Recessive |
| LCA8 | 1q31.3 | Crumbs, Drosophila, homolog of, 1 (*CRB1*) | Established | Recessive |
| LCA9 | 1p36.22 | Nicotinamide nucleotide adenyl-yltransferase 1 (*NMNAT1*) | Established | Recessive |
| LCA10 | 12q21.32 | Centrosomal protein, 290-KD (*CEP290*) | Established | Recessive |
| LCA11 | 7q32.1 | Inosine monophosphate dehydrogenase 1 (*IMPDH1*) | Established | Dominant |
| LCA12 | 1q32.3 | Retinal degeneration 3, mouse, homolog of (*RD3*) | Established | Recessive |
| LCA13 | 14p24.1 | Retinol dehydrogenase 12 (*RDH12*) | Established | Dominant |
| LCA14 | 4q32.1 | Lecithin retinol acyltransferase (*LRAT*) | Established | Recessive |
| LCA15 | 6p21.31 | Tubby-like protein 1 (*TULP1*) | Established | Recessive |
| LCA16 | 2q37.1 | Potassium channel, inwardly rectifying, subfamily J, member 13 (*KCJN13*) | Established | Recessive |
| LCA17 | 8q22.1 | Growth/differentiation factor 6 (*GDF6*) | Established | Recessive |
| LCA18 | 6p21.1 | Peripherin 2, mouse, homolog of (*PRPH2*) | Established | Recessive |

Table includes candidate genetic causes of keratoconus, and genetic causes of other corneal dystrophies that are similar to, or include keratoconus. Genetic causes of keratoconus within systemic syndromes, and corneal dystrophies that are phenotypically different to keratoconus are not listed. Eight genetic loci have been defined for keratoconus (*KTCN1-8*); however, the causative gene has not been defined in any of these loci. Two candidate genes have been identified, which are Visual System Homeobox 1 (*VSX1*) within *KTCN1* on chromosome 20p11.21, and Dedicator of Cytokinesis 9 (*DOCK9*) within *KTCN7* on chromosome 13q32, but a definite pathogenic role for these genes is not established, and for the other loci, no candidate genes have been identified. Genetic causes have been defined for several other autosomal forms of corneal defects, such as brittle cornea syndromes, corneal dystrophies and Leber congenital amaurosis (LCA), that include, or have similar clinical features to, keratoconus. LCA is a genetically heterogeneous group of early onset retinal dystrophies,^(1)^ that includes keratoconus in some patients. To date, 18 distinct genetic etiologies have been described for LCA (LCA1-18) and patients with 11 of these forms (LCA1-10 and LCA12) have been specifically reported to have keratoconus.^(2-9)^ The WES data was interrogated for novel or very rare variants these genes, that were either heterozygous in individual I.4 (with hypoparathyroidism alone) and homozygous in individual II.5 (with hypoparathyroidism and keratoconus), that would indicate an autosomal recessive inheritance, or heterozygous in both individuals, that may indicate a reduced penetrance. No rare variants were identified in the two candidate genes for keratoconus, *VSX1* and *DOCK9*. A variant that has previously been described in patients with keratoconus, c.Arg217His, was present in *VSX1*, and was heterozygous in individual I.4 without keratoconus and homozygous in individual II.5 with keratoconus. However, the role of *VSX1* and the potential pathogenicity of the Arg217His variant are disputed.^(10-12)^ Furthermore, the variant is present only in the non-canonical transcript, and is reported with a frequency of 0.21 in ExAC, with 3211 out of ~60,000 individuals (~5%) being homozygous, thus it is unlikely that this variant causes keratoconus, which occurs in about 1/2000 of the population. A novel heterozygous c.1209T>G transversion was identified in *TGFBI*, encoding a variant His403Gln in transforming growth factor beta-induced (TGFBI), which was present in both individuals (I.4 and II.5, Fig. S1*A*). No other rare (<5% MAF) or homozygous variants were present in any of the known genetic causes of corneal defects, including all 18 LCA genes. Thus, the His403Gln missense substitution, which affects an evolutionary conserved TGFBI residue and is not present in the exomes of >60,000 individuals (ExAC database), represents a potential cause of the keratoconus in this family. TGFBI is a secreted protein component of the extracellular matrix (ECM), and contains four fasciclin-1 domains and a C-terminal Arg-Gly-Asp (RGD) sequence. TGFBI has been shown to bind to several other ECM components, including collagen VI, biglycan, integrins, and fibronectin *in vitro*, and likely plays an important role in corneal structural integrity.^(13-15)^ The importance of TGFBI mutations in several forms of autosomal dominant corneal dystrophy: Reis-Bucklers corneal dystrophy, Thiel-Behnke corneal dystrophy, Groenouw type 1 corneal dystrophy (also known as granular corneal dystrophy (GCD) type 1), Avenillo corneal dystrophy (also known as GCD2) and Lattice corneal dystrophy type 3a, are well established. To date, 65 different mutations have been reported, which cluster at Arg124, or in the fourth fasciclin-1 domain of the protein, particularly at Arg555. However, the pathophysiological mechanisms of TGFBI-dependent corneal dystrophies are less well understood. Some mutations in the fourth fasciclin-1 domain alter the stability of TGFBI, such that Ala546Thr TGFBI was shown to be less stable than wild-type and formed amyloid-like fibrils in the cornea, whilst Arg555Trp TGFBI was more stable than wild-type, and formed non-amyloid deposits. This was likely due to alterations in the solvent exposure of the hydrophobic core through which monomers of TGFBI can interact with each other.^(16,17)^ By contrast, Arg124 mutations were shown not to alter the stability of TGFBI, suggesting different pathogenic mechanisms for different mutations.^(16)^ The causative role of TGFBI variants in keratoconus is less well established. To date, two TGFBI variants have been reported in patients with idiopathic keratoconus: a nonsense Gly535Stop variant in a Chinese patient, and a missense Arg533Gln variant in a Polish patient.^(18,19)^ No functional studies were reported for these variants, and their significance remains unclear. However, alterations in expression of TGFBI have been reported in corneas from patients with keratoconus but without TGFBI mutations. Thus, in regions of the cornea without scarring, TGFBI expression was decreased, whilst in areas of the cornea with scarring and amyloid deposition, TGFBI was increased and deposited, suggesting a role for TGFBI in the pathogenesis of keratoconus even in patients without TGFBI mutations.^(20,21)^

**References**

1. Elder MJ 1994 Leber congenital amaurosis and its association with keratoconus and keratoglobus. J Pediatr Ophthalmol Strabismus 31(1):38-40.

2. Coppieters F, Casteels I, Meire F, et al. 2010 Genetic screening of LCA in Belgium: predominance of CEP290 and identification of potential modifier alleles in AHI1 of CEP290-related phenotypes. Hum Mutat 31(10):E1709-1766.

3. Damji KF, Sohocki MM, Khan R, et al. 2001 Leber's congenital amaurosis with anterior keratoconus in Pakistani families is caused by the Trp278X mutation in the AIPL1 gene on 17p. Can J Ophthalmol 36(5):252-259.

4. Hameed A, Khaliq S, Ismail M, et al. 2000 A novel locus for Leber congenital amaurosis (LCA4) with anterior keratoconus mapping to chromosome 17p13. Invest Ophthalmol Vis Sci 41(3):629-633.

5. Mackay DS, Ocaka LA, Borman AD, et al. 2011 Screening of SPATA7 in patients with Leber congenital amaurosis and severe childhood-onset retinal dystrophy reveals disease-causing mutations. Invest Ophthalmol Vis Sci 52(6):3032-3038.

6. McKibbin M, Ali M, Mohamed MD, et al. 2010 Genotype-phenotype correlation for leber congenital amaurosis in Northern Pakistan. Arch Ophthalmol 128(1):107-113.

7. McMahon TT, Kim LS, Fishman GA, et al. 2009 CRB1 gene mutations are associated with keratoconus in patients with leber congenital amaurosis. Invest Ophthalmol Vis Sci 50(7):3185-3187.

8. Perrault I, Estrada-Cuzcano A, Lopez I, et al. 2013 Union makes strength: a worldwide collaborative genetic and clinical study to provide a comprehensive survey of RD3 mutations and delineate the associated phenotype. PLoS One 8(1):e51622.

9. Siemiatkowska AM, van den Born LI, van Genderen MM, et al. 2014 Novel compound heterozygous NMNAT1 variants associated with Leber congenital amaurosis. Mol Vis 20:753-759.

10. Dash DP, George S, O'Prey D, et al. 2010 Mutational screening of VSX1 in keratoconus patients from the European population. Eye (Lond) 24(6):1085-1092.

11. Shetty R, Nuijts RM, Nanaiah SG, et al. 2015 Two novel missense substitutions in the VSX1 gene: clinical and genetic analysis of families with Keratoconus from India. BMC Med Genet 16:33.

12. Tanwar M, Kumar M, Nayak B, et al. 2010 VSX1 gene analysis in keratoconus. Mol Vis 16:2395-2401.

13. Billings PC, Whitbeck JC, Adams CS, et al. 2002 The transforming growth factor-beta-inducible matrix protein (beta)ig-h3 interacts with fibronectin. J Biol Chem 277(31):28003-28009.

14. Reinboth B, Thomas J, Hanssen E, Gibson MA 2006 Beta ig-h3 interacts directly with biglycan and decorin, promotes collagen VI aggregation, and participates in ternary complexing with these macromolecules. J Biol Chem 281(12):7816-7824.

15. Son HN, Nam JO, Kim S, Kim IS 2013 Multiple FAS1 domains and the RGD motif of TGFBI act cooperatively to bind alphavbeta3 integrin, leading to anti-angiogenic and anti-tumor effects. Biochim Biophys Acta 1833(10):2378-2388.

16. Runager K, Basaiawmoit RV, Deva T, et al. 2011 Human phenotypically distinct TGFBI corneal dystrophies are linked to the stability of the fourth FAS1 domain of TGFBIp. J Biol Chem 286(7):4951-4958.

17. Koldso H, Andersen OJ, Nikolajsen CL, et al. 2015 Early Events in the Amyloid Formation of the A546T Mutant of Transforming Growth Factor beta-Induced Protein in Corneal Dystrophies Compared to the Nonfibrillating R555W and R555Q Mutants. Biochemistry 54(36):5546-5556.

18. Guan T, Liu C, Ma Z, Ding S 2012 The point mutation and polymorphism in keratoconus candidate gene TGFBI in Chinese population. Gene 503(1):137-139.

19. Karolak JA, Polakowski P, Szaflik J, Szaflik JP, Gajecka M 2015 Molecular Screening of Keratoconus Susceptibility Sequence Variants in VSX1, TGFBI, DOCK9, STK24, and IPO5 Genes in Polish Patients and Novel TGFBI Variant Identification. Ophthalmic Genet:1-7.

20. Tai TY, Damani MR, Vo R, et al. 2009 Keratoconus associated with corneal stromal amyloid deposition containing TGFBIp. Cornea 28(5):589-593.

21. Takacs L, Csutak A, Balazs E, Modis L, Jr., Berta A 1999 Expression of betaig-h3 is lower than normal in keratoconus corneas but increases with scarring. Cornea 18(5):599-605.
